# Supplementary material for: Prevalence of clinically manifested drug interactions in hospitalized patients: A systematic review and meta-analysis
Source: PLoS One. 2020 Jul 1;15(7):e0235353. doi: 10.1371/journal.pone.0235353 (PMC7329110; doi:10.1371/journal.pone.0235353)
Supplement: S1 Table — (DOCX) [file pone.0235353.s002.docx]

| **S1 Table - Complete search strategy in the searched databases.** | |
| --- | --- |
| ***PubMed*** | |
|  | Search ((("Drug Interactions"[MeSH Terms] OR "Drug Interactions"[All Fields]) OR "Drug Interaction"[All Fields]) AND ((("Clinically relevant"[All Fields] OR Real[All Fields]) OR Actual[All Fields]) OR "Adverse drug"[All Fields])) AND ((((((("hospitals"[MeSH Terms] OR ("hospitals"[MeSH Terms] OR "hospitals"[All Fields] OR "hospital"[All Fields])) OR ("hospitals"[MeSH Terms] OR "hospitals"[All Fields])) OR "inpatients"[MeSH Terms]) OR "Hospitalized patients"[All Fields]) OR "Hospitalized patient"[All Fields]) OR ("inpatients"[MeSH Terms] OR "inpatients"[All Fields] OR "inpatient"[All Fields])) OR ("inpatients"[MeSH Terms] OR "inpatients"[All Fields])) |
| ***Scopus*** | |
|  | ( ( ALL ( "Drug Interactions" ) OR ALL ( "Drug Interaction" ) ) ) AND ( ( ALL ( real ) OR ALL ( actual ) ) ) AND ( ( ALL ( hospitals ) OR ALL ( hospital ) OR ALL ( inpatient ) OR ALL ( inpatients ) OR ALL ( "hospitalized patient" ) OR ALL ( "hospitalized patients" ) ) ) AND ( LIMIT-TO ( DOCTYPE , "ar" ) ) |
| ***Web of Science*** | |
|  | Tópico: (Real) OR Tópico: (Actual) OR Tópico: ("Clinically relevant") OR Tópico: ("Adverse drug") AND Tópico: ("Drug Interactions") OR Tópico: ("Drug Interaction") AND Tópico: (Hospital) OR Tópico: (hospitals) OR Tópico: (Inpatients) OR Tópico: (Inpatient) OR Tópico:("Hospitalized patients") OR Tópico: ("Hospitalized patient") |
|  | ***Embase*** |
|  | ‘Drug interaction’ AND Hospital OR ‘Hospital patient’ AND ‘Clinically relevant’ |
|  | ***Lilacs*** |
|  | (tw:((mh:"Drug Interactions"))) OR (tw:("Drug Interactions")) OR (tw:("Drug Interaction")) AND (tw:("Clinically relevant")) OR (tw:("Actual")) OR (tw:("Real")) OR (tw:("Adverse drug")) AND (tw:((mh:"hospitals"))) OR (tw:("hospital")) OR (tw:((mh:"inpatients"))) OR (tw:("Hospitalized patients")) OR (tw:("Hospitalized patient")) OR (tw:("inpatient")) OR (tw:("hospitals")) OR (tw:("inpatients")) (tw:((tw:((mh:"Drug Interactions"))) OR (tw:("Drug Interactions")) OR (tw:("Drug Interaction")) )) AND (tw:((tw:("Clinically relevant")) OR (tw:("Actual")) OR (tw:("Real")) OR (tw:("Adverse drug")) )) AND (tw:((tw:((mh:"hospitals"))) OR (tw:("hospital")) OR (tw:((mh:"inpatients"))) OR (tw:("Hospitalized patients")) OR (tw:("Hospitalized patient")) OR (tw:("inpatient")) OR (tw:("hospitals")) OR (tw:("inpatients")))) |
