# Supplementary material for: Prevalence of clinically manifested drug interactions in hospitalized patients: A systematic review and meta-analysis
Source: PLoS One. 2020 Jul 1;15(7):e0235353. doi: 10.1371/journal.pone.0235353 (PMC7329110; doi:10.1371/journal.pone.0235353)
Supplement: S2 Table — *Studies that scored ˃ 5 stars were considered of good quality. (DOCX) [file pone.0235353.s003.docx]

| **S2 Table - Quality score of case-control studies.** | | | | | | | | | | |
| --- | --- | --- | --- | --- | --- | --- | --- | --- | --- | --- |
| **Author** | **Study design** | **Selection** | | | | **Comparability** | **Outcome** | | | **Score** |
|  |  | **Case definition adequate?** | **Representativeness of the cases** | **Selection of Controls** | **Definition of Controls** | **Based on design or analysis** | **Ascertainment of exposure** | **Same method of ascertainment for cases and controls** | **Non-Response rate** |  |
| *Muñoz-Torrero et al. 2010 | Case-control | * | * | * | - | ** | * | * | * | 8 |
| * Studies that scored ˃ 5 stars were considered of good quality | | | | | | | | | | |
