# Supplementary material for: Prevalence of clinically manifested drug interactions in hospitalized patients: A systematic review and meta-analysis
Source: PLoS One. 2020 Jul 1;15(7):e0235353. doi: 10.1371/journal.pone.0235353 (PMC7329110; doi:10.1371/journal.pone.0235353)
Supplement: Attachment — Submitted filename: Response to Reviewers.docx [file pone.0235353.s005.docx]

**Reviewer # Academic Editor:**

**1) 1. Please ensure that your manuscript meets PLOS ONE's style requirements, including those for file naming. The PLOS ONE style templates can be found at http://www.plosone.org/attachments/PLOSOne_formatting_sample_main_body.pdf and http://www.plosone.org/attachments/PLOSOne_formatting_sample_title_authors_affiliations.pdf**

*A.: Modifications were made in accordance with the reviewer’s suggestion*.

**2) Please ensure that you include a title page within your main document. We do appreciate that you have a title page document uploaded as a separate file, however, as per our author guidelines (http://journals.plos.org/plosone/s/submission-guidelines#loc-title-page) we do require this to be part of the manuscript file itself and not uploaded separately.**

**Could you therefore please include the title page into the beginning of your manuscript file itself, listing all authors and affiliations.**

*A.: Page 01 – Ok. Modifications were made in accordance with the reviewer’s suggestion*.

**3) Please kindly add your tables to be part of the manuscript and remove the uploaded table files.**

*A.: Page 09 - 14 - Ok. It was corrected.*

**4. Please consider including Forest plot representation of your data.**

*A.: We agree. The figure was inserted into the system during submission.*

**5) PLOS requires an ORCID iD for the corresponding author in Editorial Manager on papers submitted after December 6th, 2016. Please ensure that you have an ORCID iD and that it is validated in Editorial Manager. To do this, go to ‘Update my Information’ (in the upper left-hand corner of the main menu), and click on the Fetch/Validate link next to the ORCID field. This will take you to the ORCID site and allow you to create a new iD or authenticate a pre-existing iD in Editorial Manager. Please see the following video for instructions on linking an ORCID iD to your Editorial Manager account: https://www.youtube.com/watch?v=_xcclfuvtxQ**

*A.: Ok. was inserted into the system during submission.*

**6) Thank you for stating the following towards the end of your manuscript:**

**'Financial Support**

**This study was financed in part by the Coordenação de Aperfeiçoamento de Pessoal de Nível Superior - Brasil (CAPES) - Finance Code 001. The funder of the study had no role in study design, data collection, data 435 analysis, data interpretation, or writing of the report.'**

**We note that you have provided funding information that is not currently declared in your Funding Statement. However, funding information should not appear in the Acknowledgments section or other areas of your manuscript. We will only publish funding information present in the Funding Statement section of the online submission form.**

**Please remove any funding-related text from the manuscript and let us know how you would like to update your Funding Statement. Currently, your Funding Statement reads as follows:**

**'The author(s) received no specific funding for this work.'**

Ok, it was corrected. We included the amended statements within our cover letter.

**Please clarify the sources of funding (financial or material support) for your study. List the grants or organizations that supported your study, including funding received from your institution.**

**State what role the funders took in the study. If the funders had no role in your study, please state: “The funders had no role in study design, data collection and analysis, decision to publish, or preparation of the manuscript.”**

**If any authors received a salary from any of your funders, please state which authors and which funders.**

**If you did not receive any funding for this study, please state: “The authors received no specific funding for this work.”**

**Please include your amended statements within your cover letter; we will change the online submission form on your behalf.**

*A.: Thank you for all considerations. The text about Financing Statement has been removed from the manuscript. Additional, this text has been added in the cover letter and in the online submission form.*

**7) Please include captions for your Supporting Information files at the end of your manuscript, and update any in-text citations to match accordingly. Please see our Supporting Information guidelines for more information: http://journals.plos.org/plosone/s/supporting-information.**

*A.: Page 27- Modifications were made in accordance with the reviewer’s suggestion*.

**Reviewer #1**

**1) Is the manuscript technically sound, and do the data support the conclusions?**

**The manuscript must describe a technically sound piece of scientific research with data that supports the conclusions. Experiments must have been conducted rigorously, with appropriate controls, replication, and sample sizes. The conclusions must be drawn appropriately based on the data presented.**

*A.: Lines 349-352 – Thank you. Modifications were made in accordance with the reviewer’s suggestion.*

**in the method part exactly define the PICO (patient, intervention, comparator and outcome) of your study**

*A.: Lines 102-106 - Modifications were made in accordance with the reviewer’s suggestion.*

**Has the statistical analysis been performed appropriately and rigorously?**

**determine the forest plot for your study**

*A.: We agree. The figure was inserted into the system during submission.*

**Delete the references from the abstract**

*A.: Line 42- We're really sorry and thank you for the suggestion. It's fixed now..*

**Mention the limitation and bias of your study**

*A.: Lines 339-341 - Modifications were made in accordance with the reviewer’s suggestion.*
